# Immuno-Enhancing Effects of *Galium aparine* L. in Cyclophosphamide-Induced Immunosuppressed Animal Models

**DOI:** 10.3390/nu16050597

**Published:** 2024-02-22

**Authors:** Seo-yeon Lee, Seo-yeon Park, Hee-jung Park

**Affiliations:** Department Foodservice Management and Nutrition, Sangmyung University, Seoul 03016, Republic of Korea; jookyjin1@naver.com (S.-y.L.); jrphy5685@naver.com (S.-y.P.)

**Keywords:** *Galium aparine* L. extract, immune stimulation, cyclophosphamide, splenocyte proliferation, NK cell activity

## Abstract

This study investigates the immunomodulatory potential of *Galium aparine* L. (GAE) in immunodeficient animals. In this study, animals were categorized into five groups: the normal group, CYP group (cyclophosphamide intraperitoneal injection), GA5 group (cyclophosphamide + 5 μg GAE), GA50 group (cyclophosphamide + 50 μg GAE), and GA500 group (cyclophosphamide + 500 μg GAE). The CYP group exhibited significantly reduced spleen weights compared to the normal group, while the groups obtaining GAE displayed a dose-dependent increase in spleen weight. Furthermore, the GAE demonstrated dose-dependent enhancement of splenocyte proliferating activity, with significant increases observed in both LPS and ConA-induced assays. NK cell activity significantly increased in the GA50 and GA500 groups compared to the CYP group. Cytokine analysis revealed a significant increase in IL-6, TNF-α, and IFN-γ levels in ConA-induced splenocytes treated with GAE. Gene expression analysis identified 2434 DEG genes in the extract groups. Notable genes, such as Entpd1, Pgf, Thdb, Syt7, Sqor, and Rsc1al, displayed substantial differences in individual gene expression levels, suggesting their potential as target genes for immune enhancement. In conclusion, *Galium aparine* L. extract exhibits immunomodulatory properties. The observed gene expression changes further support the potential of *Galium aparine* L. extract as a natural agent for immune augmentation.

## 1. Introduction

*Galium aparine* L., a biennial herb belonging to the Rubiaceae family, is primarily characterized by flavonoid glycosides such as quercetin-galactoside, asperuloside, tannins, and other bioactive compounds. Widely distributed globally, it is traditionally used as a springtime vegetable in South Korea, and in traditional Asian medicine, it has been used for inflammatory conditions such as mumps, erysipelas, abdominal masses, and furuncle [1]. In Western folk medicine, it has been applied to treat skin disorders [2], and in certain cultures, it has been consumed in the form of tea for its purported renal and anti-obesity benefits [3,4].

Functional studies on *Galium aparine* L. have been partially conducted; it was first reported to be effective in treating ulcers in 1883 [5]. Additionally, research has explored its hepatoprotective effects [6], antioxidant properties [7], immune-modulating effect using ethanol extracts [8], anticancer effects [1], and antimicrobial activity [9]. However, studies on *Galium aparine* water extracts (GAEs) are insufficient, and specific findings regarding cellular mechanisms, gene profiles, and physiological activities related to immune function through animal experiments are limited. Therefore, further research using animal models is needed to investigate the in vivo effects of GAE on immune stimulation and to discover the related gene.

Immunosuppression refers to a state of impaired immune function, resulting in a reduced responsiveness to antigens and heightened susceptibility to disease [10]. The immune system plays a crucial role in the progression of diseases. Innate and adaptive immunity constitute defense mechanisms against external threats. Natural killer (NK) cells are part of innate immunity, exhibiting cell-killing capabilities by releasing cytotoxic granules and secreting interferon-gamma (IFN-γ) to activate macrophages and T cells [11]. Adaptive immunity, orchestrated by differentiated and matured B and T cells in secondary immune organs like the spleen and thymus, is responsible for a more specific immune response [12,13]. Various factors, such as stress, nutritional deficiencies, and aging, can lead to compromised immune function, prompting increased interest in natural dietary and medicinal interventions aimed at improving immune resilience.

Cyclophosphamide (CYP) is a substance known to induce immunosuppression and is utilized for autoimmune diseases and tolerance induction purposes [14,15]. Animal models inducing immunosuppression are commonly employed in the study of immune-regulating functions of plant-derived natural compounds [12]. Therefore, in this study, we aim to induce a mouse model with compromised immune function using CYP, examine the survival and activity of immune cells in vivo, investigate NK cell activity, and analyze the functional aspects of immune activation. Additionally, we seek to perform gene profiling at the cellular level to identify genetic biomarkers involved in immune function, with the ultimate goal of elucidating the impact of GAE on immune functionality.

## 2. Materials and Methods

### 2.1. Sample Preparation

*Galium aparine* L. was obtained from DuGen Bio (Seoul, Republic of Korea). The dried leaves were finely ground and mixed with distilled water. The resulting mixture was then combined with ten times its weight in distilled water and stirred at 4 °C for 24 h to obtain the supernatant. After filtering through a 0.22 μm filter, the obtained extract was subjected to freeze drying to yield the final extract.

### 2.2. Animals

Male Balb/c mice were obtained from Orient Bio (Seoul, Republic of Korea) and raised from 6 to 8 weeks of age. Throughout the breeding period, the diet consisted of ad libitum access to standard solid food and water, maintaining environmental conditions at a temperature of 21 ± 2 °C and a humidity of 50 ± 20%, with a light–dark cycle of 12 h/day (from 7 a.m. to 7 p.m.) and illumination ranging from 150 to 300 Lux. After acclimating the mice to the experimental environment for one week, they were categorized into five experimental groups using a random assignment method, adjusting the average body weight to approximately 21.3 g. All animal experiments were conducted in accordance with the ethical guidelines of the Institutional Review Board for Animal Experiments (NDIC, P224113).

### 2.3. Experimental Design

The experimental groups were designed as follows: control group (*n* = 3), CYP group (*n* = 3), GA5 group (*n* = 3), GA50 group (*n* = 3), and GA500 group (*n* = 3). CYP was prepared using sterile physiological saline and administered at a dosage of 80 mg/kg per animal, with a volume of 10 mL/kg for intraperitoneal injection.

A single administration of CYP via intraperitoneal injection was performed to induce immune suppression. The GAEs were administered intraperitoneally once daily for three days following CYP injection. In the case of the control group, sterile physiological saline was administered as a single intraperitoneal injection with the same 10 mL/kg volume. For the control and CYP groups, the vehicle of the GAE was intraperitoneally administered once daily for three days with the same 10 mL/kg volume. The GAEs were administered intraperitoneally at doses of 5 ug/head (GA5 group), 50 ug/head (GA50 group), and 500 ug/head (GA500 group). The CYP treatment involved a single injection on the day of experimental initiation, followed by daily injections of the test substance at the same time each day for three days. The animals were sacrificed 24 h after the final GAE treatment.

### 2.4. Blood and Immune Organ Collection

At the end of the experimental period, the mice were weighed. Blood was collected from abdominal vena cava under isoflurane anesthesia. Approximately 0.1 mL of whole blood was collected and placed in an ethylene–diamine–tetra-acetic acid (EDTA) tube for complete blood count (CBC) analysis. After blood collection, the spleen was extracted for the evaluation of immune cell proliferation and NK cell activity. The spleen was removed and weighed immediately. Finally, the organ indices were calculated as follows:

Index = weight of thymus or spleen (mg)/body weight (g) × 100 [16].

### 2.5. Proliferative Effect of Splenocytes

Splenocytes were aseptically harvested from Balb/c mice, and after adding 10 mL of Hank’s Balanced Salt Solution (HBSS, Sigma-Aldrich, St. Louis, MO, USA) to the cell culture dish, the cells were homogenized using a 40 μm nylon cell strainer (BD Biosciences, San Jose, CA, USA). The obtained splenocytes were processed on a Histopaque (Sigma-Aldrich, St. Louis, MO, USA) gradient to separate lymphocytes, washed, then dispersed in culture medium (RPMI 1640, 10% FBS, 100 U/mL penicillin, 100 mg/mL streptomycin) at a concentration of 3.0 × 10^6^ cells/mL. The cells were dispensed into a 96-well plate (Nunc, Rosklide, Denmark) with 100 μL in each well for measurement of cell proliferation. As a positive control for T- and B-cell mitogens, concanavalin-A (ConA, Sigma-Aldrich, St. Louis, MO, USA) and lipopolysaccharide (LPS, Sigma-Aldrich, St. Louis, MO, USA) were treated at final concentrations of 5 ug/mL each. The control group received an equal volume of culture medium. The cells were then cultured in a 37 °C, 5% CO_2_ incubator. After 48 h of cultivation, the absorbance was measured at 450 nm using a microplate reader to compare the cell activity. The proliferative activity of splenocytes was assessed using a colorimetric assay (Ez-Cytox, Dugen, Seoul, Republic of Korea).

### 2.6. NK Cell Activity against YAC-1 Cells

To assess the cytotoxicity of NK cells, a modified lactate dehydrogenase (LDH) release assay was employed, where NK cells attacked and destroyed YAC-1 cells, a type of cancer cell (NK-sensitive cell line). YAC-1 cells were adjusted to a concentration of 1 × 10^4^ cells/100 μL in a 96-well, U-bottomed culture plate (Corning Glass Works, Corning, NY, USA). NK cells were then isolated from the splenocytes using an NK cell isolation kit (Miltenyi Biotec, Bergosch Gladbach, Germany). Single-cell suspensions of NK cells were added to YAC-1 cells (1 × 10^4^ cells/well) to achieve an effector-to-target cell ratio of 5:1 or 2.5:1 in a U-bottomed 96-well plate, and the cultures were incubated for 6 h. YAC-1 lymphoma cells were cultured in RPMI-1640 (Gibco BRL, Thermo Fisher, Grand Island, NY, USA) supplemented with 7.5% FBS and L-glutamine.

After incubation, the cells were centrifuged, and 100 μL of the supernatant containing released LDH was transferred to flat-bottom microplates (Nunc, Roskilde, Denmark). The LDH release assay utilized an ELISA system (microtiter plate reader, Roche, Mannheim, Germany). Initially, LDH substrate mixture (100 μL) was added, and the reaction was allowed to proceed in the dark at room temperature (15–25 °C) for 30 min. The reaction was then stopped by adding 50 μL of 1 N HCl, and absorbance at 490 nm was measured. For spontaneous LDH measurement, only culture medium was added to control wells, and for maximum LDH release (total lysis) determination, Triton X-100 solution (high control) was added to completely lyse the cells. The percentage of cytotoxicity was calculated using the following formula based on the LDH released from each culture:

Cytotoxicity (%) = (Experimental LDH release−Spontaneous LDH release/Maximum LDH release−Spontaneous LDH release) × 100.

### 2.7. Splenocyte Proliferation Cytokine

The splenocyte suspension was distributed onto a 96-well microplate at 200 μL/well and cultured at 37 °C for 72 h in a 5% CO_2_ incubator. In the culture supernatants, IFN-γ, interleukin (IL)-6, and tumor necrosis factor (TNF)-α were measured using a commercial ELISA kit (R&D Systems, Minneapolis, MN, USA).

### 2.8. RNA-seq

Raw 264.7 cells were cultured in Dulbecco’s Modified Eagle’s Medium (DMEM, Sigma-Aldrich, St. Louis, MO, USA) supplemented with 10% fetal bovine serum (FBS), 100 U/mL penicillin, and 100 ug/mL streptomycin. Raw 264.7 cells were seeded at 4× 10^4^/well in a 96-well plate containing DMEM and incubated for 24 h. Then, the cells were divided into groups with and without GAE added, along with LPS, and cultured in DMEM for 48 h. Total RNA was extracted from each sample using RNeasy kit (Qiagen, Hilden, German) reagent following the manufacturer’s instructions, and the RNA samples were submitted to Macrogen Corporation (Seoul, Republic of Korea) for RNA sequencing (RNA-seq). Sequencing libraries were prepared by randomly fragmenting cDNA samples, then ligating 5′ and 3′ adapters. The library was loaded onto a flow cell with complementary surface binding to the library adapter, and each fragment was amplified to complete clustering. After clustering was completed, sequencing was performed using the Illumina platform. Whole-genome sequencing resulted in the sequencing of a total of 7,652,124,004 bases. After sequencing, quality control analysis of raw reads was performed, producing basic statistics such as overall read quality, total bases, total reads, and GC (%). To reduce bias in the analysis results, a preprocessing process was performed to remove artifacts such as low-quality or adapter sequences, contaminant DNA, and PCR duplicates, followed by mapping to the reference genome using the HISAT2 program and generation of aligned reads. Transcript assembly was performed using the StringTie program using reference-based aligned reads information. Fragments per kilobase of transcript per million mapped reads (FPKM)/reads per kilobase of transcript per million mapped reads (RPKM) is a normalization value considering the read count, transcript length, and depth of coverage of the expression level obtained through transcript quantification of each sample and transcripts per kilobase million (TPM) values to extract the expression profile. Using this value, differentially expressed gene (DEG) analysis was performed using edgeR for the three comparison combinations, and genes that satisfied the conditions of |fc| ≥ 2 and exactTest raw *p*-value < 0.05 were extracted. A total of 2434 genes were identified. In cases where known genetic information was available, functional annotation and gene-set enrichment analysis based on the Gene Ontology (GO) and Kyoto Encyclopedia of Genes and Genomes (KEGG) databases were performed, targeting differentially expressed genes.

### 2.9. Statistical Analysis

All obtained experimental results are presented as mean ± standard deviation, and statistical analyses were performed using SPSS (version 20, IBM SPSS Statistics, Chicago, IL, USA). Levene’s test was conducted to assess the homogeneity of variances for all data. In cases where homogeneity of variances was observed, a one-way analysis of variance (ANOVA) was employed. Upon observing significance, the least significant difference (LSD) test was applied for post hoc analysis to identify significant differences between the control group and experimental groups. In the presence of heteroscedasticity, Dunnett’s T3 test was used for post hoc analysis. The significance level was set at two-tailed 5% and 1%. It should be noted that, depending on the circumstances, appropriate statistical analysis methods may be adjusted and applied.

## 3. Results

### 3.1. Changes in Mouse Body Weight, Spleen Weight, and Spleen Cell Index

The spleen, the largest secondary lymphoid organ found in vertebrates, plays a crucial role in the body’s immune response through the T cell-rich region called the periarteriolar lymphoid sheaths and the B cell-rich zone within white pulp [17]. In this study, while there were no significant changes in body weight based on the experimental groups (Table 1), the group treated with CYP showed a tendency of about a 5% decrease in body weight. The spleen weight exhibited a similar pattern to that of body weight. The spleen organ index was significantly lower in the CYP group, and there was a concentration-dependent increase in the spleen organ index with varying concentrations of GAE. In the G5 group, the spleen organ index recovered to a level similar to that of the control group. This suggests that Galium aparine may partially counteract the immunosuppressive effects of CYP.

### 3.2. WBC Number and WBC Differential Count

**Figure 1:**
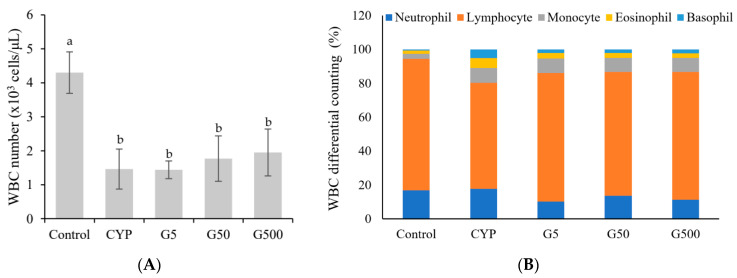
Effect of *Galium aparine* extracts on white blood cell differential count and composition (%) in cyclophosphamide-treated mice. Control group (saline), CYP: cyclophosphamide injection; G5 group: 5 mg/head GAE injection; G50 group: 50 mg/head GAE injection; G500 group: 500 mg/head GAE injection. (**A**) WBC number. (**B**) WBC differential counting. The data are expressed as the mean ± SD, showing significant differences (*p* < 0.05) with different letters among the 5 groups.

White blood cells (WBCs), or leukocytes, are composed of five main types in normal individuals: neutrophils, eosinophils, basophils, monocytes, and lymphocytes. Their primary function is to perform immune functions, blocking external infections. The differential count of white blood cells assesses whether the distribution of these cell types is normal or if there is an increase (leukocytosis) or decrease (leukopenia) in a specific cell type. Monocytes are cells that fight against specific infections, while lymphocytes regulate antibody production and participate in immune responses. Neutrophils combat bacterial or fungal infections. Eosinophils are involved in cellular immune responses, particularly during parasitic infections or allergic diseases. Basophils play a role in regulating tissue damage and inflammation. In this experiment, the total white blood cell counts significantly decreased in mice treated with CYP compared to the control group (Figure 1). Specifically, the lymphocyte counts significantly decreased in the CYP-treated group, but they recovered in the *Galium aparine* extract-treated group. The proportion of eosinophils and basophils significantly increased in the CYP-treated group. However, upon GAE treatment, eosinophils returned to control levels, while basophils showed partial recovery.

### 3.3. Splenocyte Proliferation

**Figure 2:**
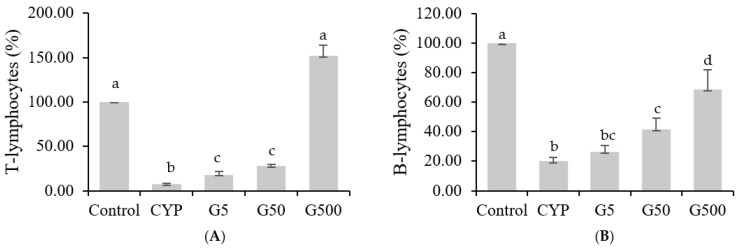
Effects of *Galium aparine* extracts on T and B cell proliferation in splenocytes of Balb/c mice immunosuppressed by cyclophosphamide. (**A**) T-lymphocyte proliferation. (**B**) B-lymphocyte proliferation. The data are expressed as the mean ± SD, showing significant differences (*p* < 0.05) with different letters among the 5 groups.

The spleen acts as a secondary lymphoid organ that filters blood, removes antigens from the bloodstream, and initiates adaptive immune responses by activating immune cells. The spleen encompasses various immune cells, including T lymphocytes, B lymphocytes, macrophages, and dendritic cells. Since T cells and B cells continuously circulate through the blood to and from the spleen, changes in the number of splenic cells are considered reflective of alterations in lymphocytes [18]. CYP alkylation of DNA in splenic cells inhibits immune responses mediated by T and B lymphocytes [19]. This study aimed to evaluate the impact of *Galium aparine* extract on CYP-suppressed splenic immune cell responses. The results showed a significant decrease in splenic cell proliferation in the CYP group compared to the control group when treated with Con A. However, the injection of GAE restored splenic cell proliferation to levels comparable to the those of the control group (Figure 2A). In the case of LPS treatment, CYP injection led to a significant decrease in splenocyte proliferation compared to the control group, while GAE injection showed a dose-dependent increase in proliferation (Figure 2B). Compared to the CYP group, GAE treatment resulted in higher proliferation of T lymphocytes and B lymphocytes, suggesting that the extract stimulates the development of immune organs and cells in immune-suppressed mice, enhancing the immune system.

### 3.4. NK Cell Activity

**Figure 3:**
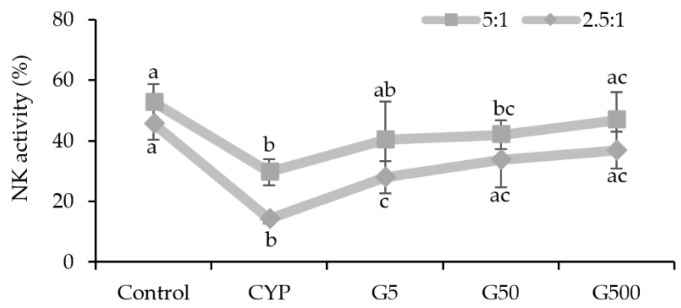
Effects of *Galium aparine* extracts on natural killer cell activity against Yac-1 (effector cell: YAC-1 = 5:1 or 2.5:1) in the splenocytes of mice immunosuppressed by cyclophosphamide. Control (saline), CYP: cyclophosphamide injection; G5 group: 5 mg/head GAE injection; G50 group: 50 mg/head GAE injection; G500 group: 500 mg/head GAE injection. The data are expressed as the mean ± SD, showing significant differences (*p* < 0.05) with different letters among the 5 groups.

NK cells are recognized as a crucial component of the innate immune system [20]. NK cells are known to seek and eliminate infected or cancerous cells, as well as to activate other phagocytic cells by secreting soluble factors, including cytokines, to protect the host [20,21]. In this study, the impact of GAE on NK cell activity in the spleen of immunocompromised mice was analyzed. The results revealed heightened NK cell activity in all GAE treatment groups, with percentages of 40.3%, 41.8%, and 46.6% for different concentrations, compared to 29.6% in the CYP group and 52.6% in the control group (Figure 3). This increase in NK cell activity indicates that GAE has the potential to restore the diminished NK cell activity caused by CYP. Therefore, *Galium aparine* extract is considered to enhance immunity by regulating innate immune responses in immunocompromised conditions.

### 3.5. The Impact of Galium Aparine Extract on Cytokine Levels

**Figure 4:**
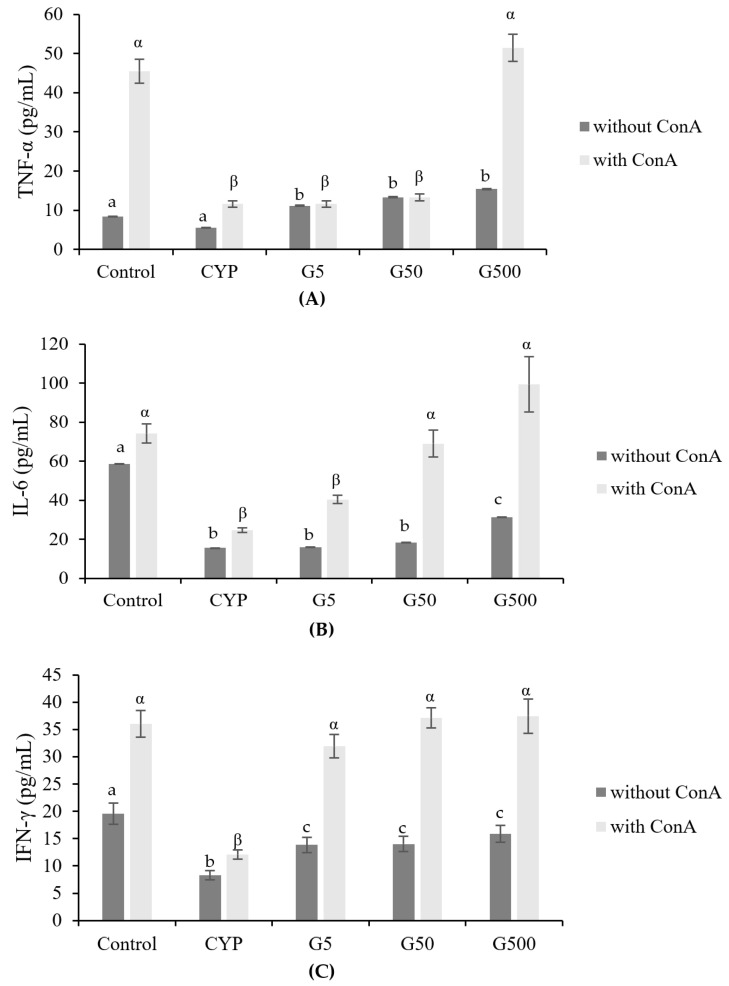
Effects of *Galium aparine* extracts on TNF-α, IL-6, and IFN-γ production from ConA-stimulated primary splenocytes prepared from cyclophosphamide-treated mice. (**A**) TNF-α. (**B**) IL-6. (**C**) IFN-γ. The data are expressed as the mean ± SD, showing significant differences (*p* < 0.05) with different letters among the 5 groups for each ConA treatment condition.

CYP is known to reduce the number of T lymphocytes and influence the secretion of cytokines, proteins secreted by immune cells that play a crucial role in signaling to target cells and regulating immune responses [22,23]. Immunodeficiency induced by CYP treatment has been associated with a decrease in the production of cytokines such as TNF-α and IL-6. GAE was found to rejuvenate the activity of Th1- or Th17-related immune response (Figure 4). Specifically, the levels of TNF-α were reduced in the CYP group compared to the control group but were restored in the GA500 group. Similarly, IL-6 levels decreased in the CYP group compared to the control group, showing a concentration-dependent increase with GAE. IFN-γ followed a pattern similar to that of IL-6, with recovery observed even in the GAE group. Therefore, *Galium aparine* extract may influence the regulation of Th1 cytokine secretion, and its administration could potentially enhance immunity by modulating the levels of these cytokines.

### 3.6. Identification of Differentially Expressed Genes (DEGs) in the GAE Group

**Figure 5:**
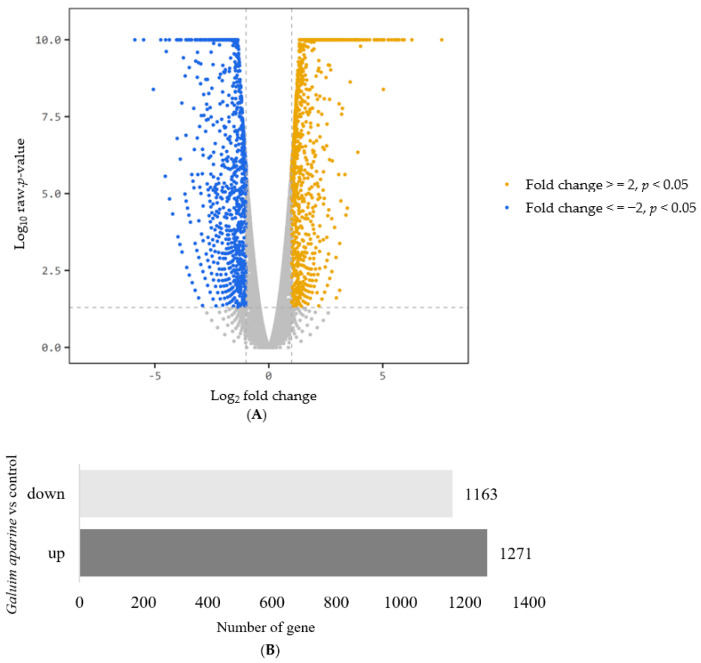
Changes in mRNA expression. (**A**) Volcano plot (**B**) The number of up-regulated and down-regulated genes. |Fold change| ≥ 2, *p* < 0.05.

Based on the criteria of fold change ≥2, as well as a *p* value < 0.05, there were 2434 mRNAs that were differentially expressed between the treatment groups. Among them, 1271 genes were up-regulated, and 1163 genes were down-regulated, which are shown on a volcano plot in Figure 5.

**Figure 6:**
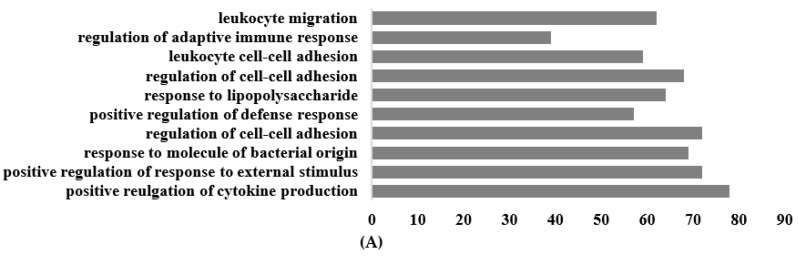

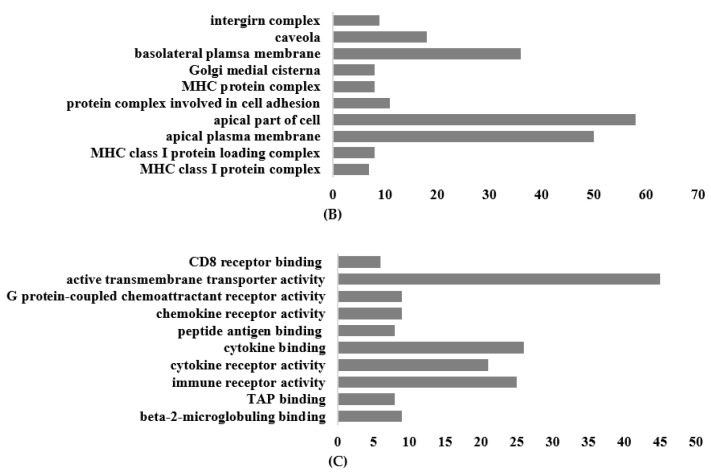
Top 10 terms of GO functional analysis between *Galium aparine* and the control. (**A**) Biological process. (**B**) Cellular component. (**C**) Molecular function.

The most significantly enriched Gene Ontology (GO) annotations in the classification of biological processes, cellular components, and molecular function among the differentially expressed mRNAs are shown in Figure 6. For the biological process, the primary enriched GO terms included positive regulation of cytokine production, positive regulation of response to external stimulus, response to molecules of bacterial origin, regulation of cell–cell adhesion, positive regulation of defense response, and regulation of adaptive immune response. In terms of molecular function, the genes exhibited activities related to active transmembrane transporter activity, cytokine binding, cytokine receptor activity, and immune receptor activity (Figure 6A).

Among the genes showing differential expression, there were 20 genes with a fold change of |30| or higher (Table 2). Up-regulated DEGs were ectonucleoside triphosphate diphosphohydrolase 1 (Entpd1); placental growth factor (Pgf); synaptotagmin VII (Syt7); thrombomodulin (Thbd); CD93 antigen (Cd93); regulatory solute carrier protein (Rsc1a1); mitochondria-localized glutamic acid-rich protein (Mgarp); leukotriene B4 receptor 1 (Ltb4r1); selenium binding protein 1 (Selenbp1); prostaglandin E receptor 2 (Ptger2); ATPase, class II, type 9A (Apt9a); GLI pathogenesis-related 2 (Glipr2); transmembrane and immunoglobulin domain-containing 3 (Tmigd3); G protein-coupled receptor 31 (Gpr31b); Fc receptor, IgG, high affinity I (Fcgr1); and selenium binding protein 2 (Selenbp2). The down-regulated genes include small nucleolar RNA, H/ACA box 73a (Snora73a); RAN binding protein 3-like (Ranbp31); and RAB15 (Ras oncogene family member Rab15), among others.

## 4. Discussion

The impairment of the immune system through immunosuppression alleviates the body’s ability to combat specific infections. Exploring immunomodulatory agents derived from natural ingredients has become a focal point in addressing diseases characterized by immunosuppression.

*Galium aparine* has traditionally been used as a natural material for its anti-inflammatory and antioxidant properties [7,8], but specific studies using animal models are insufficient. In this study, we directly investigated immunomodulatory effects through animal experiments and aimed to identify target genes for immune enhancement therapy through involved genes.

The model group treated with CYP exhibited a significant decrease in weight, WBC count, and splenocyte index compared to the normal group. Excessive CYP administration is known to reduce the count of white blood cells, particularly lymphocytes. Generally, immune stimulants can enhance the weight of immune organs and the proliferation of immune cells [24,25]. GAE showed a protective effect against CYP-induced immunosuppression, recovering weight and WBC composition. The spleen functions as a crucial immune organ, representing the secondary lymphatic tissue [26,27]. It plays a role in both immune responses and hematopoiesis [28,29], contributing to the immune defense against mycobacterial infections [30]. Comprising diverse immune cells like macrophages, monocytes, B and T cells, and NK cells, each with distinct immune functions, the spleen modulates immune responses [31,32]. GAE led to an improvement in the spleen index. Administration of 50 and 500 mg/head of GAE significantly improved the CYP-induced proliferation of splenic lymphocytes, indicating the role of GAE in the activation of T and B lymphocytes. GAE demonstrated a mitogenic effect, directly promoting the proliferation of splenic cells. This outcome suggests that GAE enhances the immune system.

Lymphocytes consist primarily of NK cells, T cells, and B cells. Among these, NK cells are renowned for their nonspecific elimination of pathogens and infected cells, contributing significantly to the early phases of the immune system’s infection control [33,34]. NK cells serve as a frontline defense against invasion and infection by rapidly targeting and destroying both abnormal and virus-infected cells without the need for prior sensitization [35,36,37,38]. The pivotal role of NK cells in pathogen-induced immune responses and tumor growth control has been suggested based on this information [39,40]. NK cells have demonstrated their ability to modulate immune responses by releasing immune-regulating cytokines and chemokines [41,42], influencing dendritic cell (DC) activity and affecting granulocyte growth and differentiation [42,43,44]. In summary, NK cells, classified as innate immune cells with cytotoxicity against tumor and virus-infected cells, also secrete signaling substances like IFN-γ, TNF-α, and chemokines. Through these actions, NK cells play a crucial role in innate immune responses, serving as a bridge from innate to adaptive immune responses [45,46]. NK cell activity was significantly lower in the CYP group than in the control group. Here, GAE simultaneously increased NK cell activity in immunosuppressed mice treated with CYP. In conclusion, these results support the idea that GA extract can enhance host immunity, regardless of whether the host is in a normal or weakened state. Therefore, GAE is suggested to induce effective defense against antigens by increasing the number of effector cells that respond to antigen exposure.

Macrophages play a pivotal role in the innate immune system, serving as a crucial defense mechanism against host infections and participating in the elimination of tumor cells. The exploration of various biological response modifiers for their potential to modulate the antitumor properties of macrophages is an active area of interest in cancer chemotherapy closely linked to immunomodulatory activities [47]. Macrophages become activated through exposure to IFN-γ and microbial stimuli, such as LPS [48]. Our findings indicate that CYP treatment led to impaired phagocytosis by peritoneal macrophages. However, treatment with GAE exhibited a dose-dependent enhancement of macrophage activity in immunosuppressed mice. Pro-inflammatory cytokines, such as TNF-α and IL-6, activate NF-κB. TNF-α is primarily secreted by activated macrophages and plays a crucial role in inducing inflammation and apoptosis, making it an important cytokine in the regulation of immune cell activity [49]. In this study, we observed a reduction in the production of TNF-α in the immunosuppressed model, which was restored to normal control levels upon GAE administration. Additionally, a significant increase in the production of IL-6, which is elevated by TNF-α, was confirmed to follow a pattern similar to that of TNF-α, reaching significantly higher levels in the GAE treatment group. Yoon et al. reported that GAE regulates the secretion of cytokines such as TNF-α and IL-6, suggesting its immunomodulatory activity, with a particular emphasis on the role of compounds aiding in this function as single compounds. Normally, Th1 cells release IFN-γ, IL-2, and TNF-α, which mainly contribute to cell-mediated immune response [50,51]. GAE holds promise as a valuable immunomodulatory agent for boosting immune function in compromised states, showcasing its potential utility in immune enhancement.

In this study, *Galium aparine* resulted in significant changes in the expression of multiple genes associated with the immune-modulating mechanism. In cancer treatment, genes Entpd1 [52], Pgf [53], and Selenbp1 [54] are being utilized as immunomodulatory targets. Ptger2 is a gene involved in mast cell activation [55], while Syt7 regulates activation in mast cells and dendritic cells [56]. Thbd and Cd93 are known as genes that influence innate immune responses [57,58]. Ltb4r1 plays a role in the immune response, particularly affecting the numbers of T cells, especially CD8 [59]. Mgarp is a gene associated with immune functions related to mitochondria [60]. Rab15 is involved in positive regulation of regulated secretory pathway-associated immunodeficiency [61]. The Fcgr1 gene encodes a protein that plays an important role in the immune response, acting as a high-affinity Fc-gamma receptor [62]. Upon examining the KEGG pathway using these genes, the cAMP signaling pathway, anti-inflammatory response, inflammatory mediator regulation of TRP channels, and FcγR-mediated phagocytosis pathways in cancer were included. The current study revealed that GAE regulated the gene expression profile involving the immune function. Deciphering the precise molecular mechanisms of the functions of these coding RNAs in immune enhancement is vital for the exploration of new potential targets for therapy.

There are several limitations of this study that should be noted. The main limitation was that the test substance was administered through injections rather than chronic administration. A chronic administration experiment will be carried out in the future to elucidate potential supplementation. In addition, the primary active components and potential interactions with other medications remain unclear and necessitate further investigation. This experiment was conducted using only crude extracts, and there is a need for further research to identify functional components. Lastly, the current data on gene expression are derived from microarray analysis, and the expression levels of differentially expressed RNAs have not been verified. These analyses aimed to provide insights into the functional roles of genes that are differentially expressed between the two groups and offer a basis for further investigation.

## 5. Conclusions

This study presents, for the first time, evidence that GAE injection leads to a dose-dependent acceleration of recovery from immunosuppression in cyclophosphamide-treated mice. The potential application of GAE as an effective adjuvant therapy for immunomodulation is substantial. Our findings offer experimental evidence to support future research and the clinical utilization of GAE injection. The findings reported in the present study provide insight into genes and their molecular mechanisms governing differences in immune-enhancing capacity.

## Figures and Tables

**Table 1:** Effects of Galium aparine on body weight, spleen weight, and organ index in the experimental model.

Group	Body Weight (g)		Spleen Weight (g)	Spleen Organ Index
	Initial	Final		
Control	21.93 ± 0.93	22.80 ± 0.85	0.0856 ± 0.010	0.37 ± 0.03
CYP group	21.90 ± 0.96	20.90 ± 1.37	0.0497 ± 0.002	0.24 ± 0.02 **
G5 (CYP + GA 5 ug/head)	21.97 ± 0.86	21.17 ± 1.22	0.0573 ± 0.005	0.27 ± 0.02 **
G50 (CYP + GA50 ug/head)	21.83 ± 0.99	21.27 ± 1.23	0.0575 ± 0.008	0.27 ± 0.03 **
G500 (CYP + GA500 ug/head)	21.77 ± 1.01	21.07 ± 0.76	0.0648 ± 0.003	0.31 ± 0.02

The data are expressed as means ± SD. ** *p* < 0.05 compared with the saline-treated control group.

**Table 2:** Differentially expressed mRNAs with *Galium aparine*.

Gene_Symbol	Description	Fold Change	*p* Value
Entpd1	ectonucleoside triphosphate diphosphohydrolase 1	192.001412	8.53 × 10^−104^
Pgf	placental growth factor	77.296374	3.53 × 10^−78^
Syt7	synaptotagmin VII	61.092434	8.08 × 10^−85^
Thbd	thrombomodulin	57.921141	1.90 × 10^−29^
Cd93	CD93 antigen	51.973585	3.28 × 10^−43^
Rsc1a1	regulatory solute carrier protein, family 1, member 1	49.77405	7.07 × 10^−58^
Mgarp	mitochondria localized glutamic acid rich protein	49.049362	1.15 × 10^−55^
Ltb4r1	leukotriene B4 receptor 1	45.572534	1.70 × 10^−33^
Selenbp1	selenium binding protein 1	42.026476	2.07 × 10^−65^
Ptger2	prostaglandin E receptor 2 (subtype EP2)	38.651755	1.06 × 10^−53^
Atp9a	ATPase, class II, type 9A	37.514388	1.96 × 10^−41^
Glipr2	GLI pathogenesis-related 2	33.120953	1.47 × 10^−74^
Tmigd3	transmembrane and immunoglobulin domain containing 3	32.502061	3.77 × 10^−18^
Gpr31b	G protein-coupled receptor 31, D17Leh66b region	32.487318	8.17 × 10^−8^
Fcgr1	Fc receptor, IgG, high affinity I	32.281222	1.09 × 10^−85^
Selenbp2	selenium binding protein 2	31.256647	8.50 × 10^−28^
Snora73a	small nucleolar RNA, H/ACA box 73a	−33.49652262	8.17 × 10^−08^
Ranbp3l	RAN binding protein 3-like	−45.13830628	3.08 × 10^−47^
Rab15	RAB15, member RAS oncogene family	−58.7506144	9.63 × 10^−79^

The above genes represent those out of 2434 expressing a fold change of |30| or more when comparing between groups.

## Data Availability

Data are contained within the article.
